# Diet and Cell Size Both Affect Queen-Worker Differentiation through DNA Methylation in Honey Bees (*Apis mellifera*, Apidae)

**DOI:** 10.1371/journal.pone.0018808

**Published:** 2011-04-26

**Authors:** Yuan Yuan Shi, Zachary Y. Huang, Zhi Jiang Zeng, Zi Long Wang, Xiao Bo Wu, Wei Yu Yan

**Affiliations:** 1 Honeybee Research Institute, Jiangxi Agricultural University, Nanchang, Jiangxi, China; 2 Department of Entomology, Michigan State University, East Lansing, Michigan, United States of America; 3 Ecology, Evolutionary Biology and Behavior Program, Michigan State University, East Lansing, Michigan, United States of America; Oregon State University, United States of America

## Abstract

**Background:**

Young larvae of the honey bee (*Apis mellifera*) are totipotent; they can become either queens (reproductives) or workers (largely sterile helpers). DNA methylation has been shown to play an important role in this differentiation. In this study, we examine the contributions of diet and cell size to caste differentiation.

**Methodology/Principal Findings:**

We measured the activity and gene expression of one key enzyme involved in methylation, Dnmt3; the rates of methylation in the gene *dynactin p62*; as well as morphological characteristics of adult bees developed either from larvae fed with worker jelly or royal jelly; and larvae raised in either queen or worker cells. We show that both diet type and cell size contributed to the queen-worker differentiation, and that the two factors affected different methylation sites inside the same gene *dynactin p62*.

**Conclusions/Significance:**

We confirm previous findings that Dnmt3 plays a critical role in honey bee caste differentiation. Further, we show for the first time that cell size also plays a role in influencing larval development when diet is kept the same.

## Introduction

The honey bee (*Apis mellifera*) is a highly eusocial insect and is characterized by its sophisticated division of labor, dance communication for efficient use of resources, and its high degree of cohesion, functioning as a “superorganism” [1]. A normal honey bee colony is made up of a single reproductive queen, from a few to some thousands of haploid drones (dependent on season), and tens of thousands of non-reproductive female workers. Even though both the queen and her workers are genetically identical, the queen is the reproductive member and activates her ovaries shortly after mating, whereas workers have a highly reduced number of ovarioles and can only produce a limited number of eggs when activated (under queenless conditions). Besides ovariole numbers, queens and workers exhibit vast differences in morphology, behavior, physiology and longevity [2]–[4].

The mechanisms for differentiation between queen and worker were a hot topic in the 1950s [5]. These earlier studies established that worker larvae up to 3 days after hatching are totipotent and can still develop into virtually normal queens if they are transferred to queen cells and raised inside the colony. After 3.5 days, only intercastes can develop. This suggests that at this age, larval development is largely fixed (no queens can be produced), but also queen and worker represents the extremes of a continuum (not all become workers). Queen cells are much larger than worker cells, and are vertically oriented while worker cells are oriented horizontally. Young workers with developed hypopharyngeal and mandibular glands (“nurses”) detect the differences between worker and queen cells and feed the larvae different food [6]. Larvae in queen cells are provisioned with royal jelly (RJ), while those in worker cells are provided with worker jelly (WJ). The consensus now is that either different sugar concentrations or other phagastimulants in RJ enables queen larvae to eat more food, which, perhaps through stretch receptors in the gut, sends signals to the brain [7]. Higher juvenile hormone (JH) synthesis by the *corpora allata* results in reduced apoptosis and the expression of queen-specific genes, resulting in the queen phenotype [8]. Queen and worker larvae have very different activated genes [9], as well as protein profiles [10].

The most recent discovery is that DNA methylation is implicated in caste determination. Wang et al. [11] first established that honey bees have a DNA methylation system with two active orthologs of vertebrate DNA methyltransferases, Dnmt1 and Dnmt3 [11]. Dnmt3 was then shown to be involved in caste determination in honey bees [12]. Silencing this gene in newly hatched larvae results in reduced rates of methylation, which leads to a significantly higher proportion of queens. Reduced methylation therefore appears to mimic the effect of royal jelly (RJ). Kucharski et al. [12] also determined the percentage of methylation at 10 CpG sites in *dynactin p62*, a gene that responds to dietary changes in *Drosophila*. Again, injection of double-stranded Dnmt3 RNA resulted in decreased rates of methylation in *dynactin p62*, when the 10 CpG sites are considered together. This mimics the high methylation rates in worker larvae, and low methylation rates in queen larvae, when reared inside colonies.

While the effects of nutrition on caste determination have been well studied [10], [12], [13], [14], whether the size difference between worker and queen cells also contributes to caste determination is not clear. In this study we report the effects of both nutrition and cell-size on Dnmt3 activity, its gene expression, and the resulting phenotype of adult bees. We also tested the hypothesis that nutrition and cell-size affect the methylation of different CpG sites in the *dynactin p62*.

## Results

**Figure 1:**
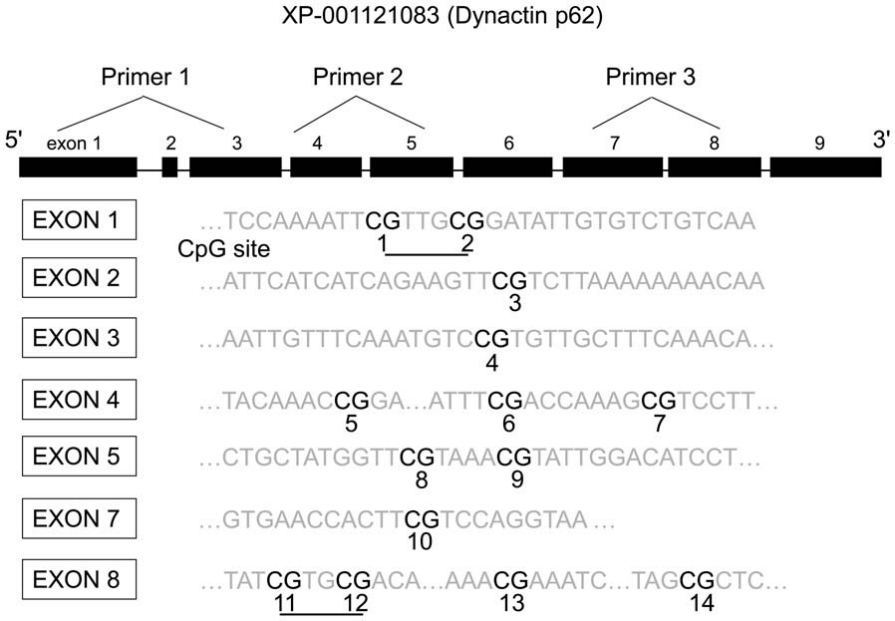
Schematic diagram showing primer locations, exons (top) and CpG sites within the various exons (bottom) in the gene *dynactin p62*. Polymerase chain reaction primers were designed to cover most of the CpG sites, except those in exons 6 and 9. Numbers 1–14 refer to locations of the CpG sites, and underlining highlights those units with two CpG sites tested at the same time. For CpG 9 and 13, we did not detect any methylation and they were not reported in Table 1.

Individual CpG sites distributed among nearly all exons of the gene *dynactin p62* are shown in Fig. 1. Two sites (CpG9 and 13) were not detected to have any methylation and are not presented in Table 1. Two locations (CpG1,2, CpG13,14) had two CpG sites too close to be separated and were reported as a single site in Table 1.

**Table 1:** Percentage of methylation in *dynactin p62* (Mean±SE, after arcsin transformation) as affected by food type (A) and cell type (B).

Treatment	CpG-1,2	CpG-3	CpG-4	CpG-5	CpG-6	CpG-7	CpG-8	CpG-10	CpG-11,12	CpG-14
**A. Effect of food type on queen-worker differentiation (6 day old larvae)**										
Days fed RJ: 5	1.9±1.8 a	1.4±0.3 a	**76.6±0.7 a**	**75.7±4.6 a**	13.4±1.5 a	12.5±2.1 a	**70.2±0.9 a**	80.2±1.3 a	21.3±5.7 a	**67.8±2.3 a**
Days fed RJ: 4	2.3±1.5 a	2.0±0.5 a	**81.5±0.8 b**	**80.1±4.5 b**	14.6±1.9 a	13.9±1.9 a	**76.1±1.3 b**	82.5±1.1 a	23.2±5.7 a	**72.1±2.1 b**
Days fed RJ: 3	2.5±1.6 a	2.6±0.2 a	**86.8±0.5 c**	**85.1±4.4 c**	15.9±1.7 a	14.1±2.2 a	**82.6±1.1 c**	82.9±0.9 a	24.7±5.2 a	**78.8±2.6 c**
**B. Effect of cell type on queen-worker differentiation**										
**3 day old larvae**										
Queen-cell	**2.9±1.3 a**	1.1±1.2 a	**70.3±1.0 a**	74.6±2.2 a	34.8±2.1 a	**45.9±1.3 a**	75.1±0.9 a	**75.1±2.2 a**	33.1±1.9 a	63.0±1.4 a
Worker-cell	**6.2±1.4 b**	5.9±1.4 a	**78.2±1.1 b**	74.3±2.0 a	35.5±2.3 a	**54.8±1.1 b**	77.5±1.1 a	**85.9±2.4 b**	34.8±2.1 a	65.2±1.6 a
**5 day old larvae**										
Queen-cell	**3.2±0.6 a**	**3.1±0.7 a**	**72.5±1.5 a**	80.1±2.2 a	24.3±1.3 a	**45.8±2.4 a**	**79.3±2.1 a**	**76.9±0.8 a**	26.7±1.2 a	71.2±2.2 a
Worker-cell	**8.9±0.7 b**	**8.5±0.9 b**	**81.0±1.6 b**	82.9±2.1 a	25.1±1.1 a	**63.3±2.0 b**	**88.9±1.9 b**	**85.7±0.6 b**	28.9±1.1 a	72.3±1.9 a

### Effect of diet on caste differentiation

**Figure 2:**
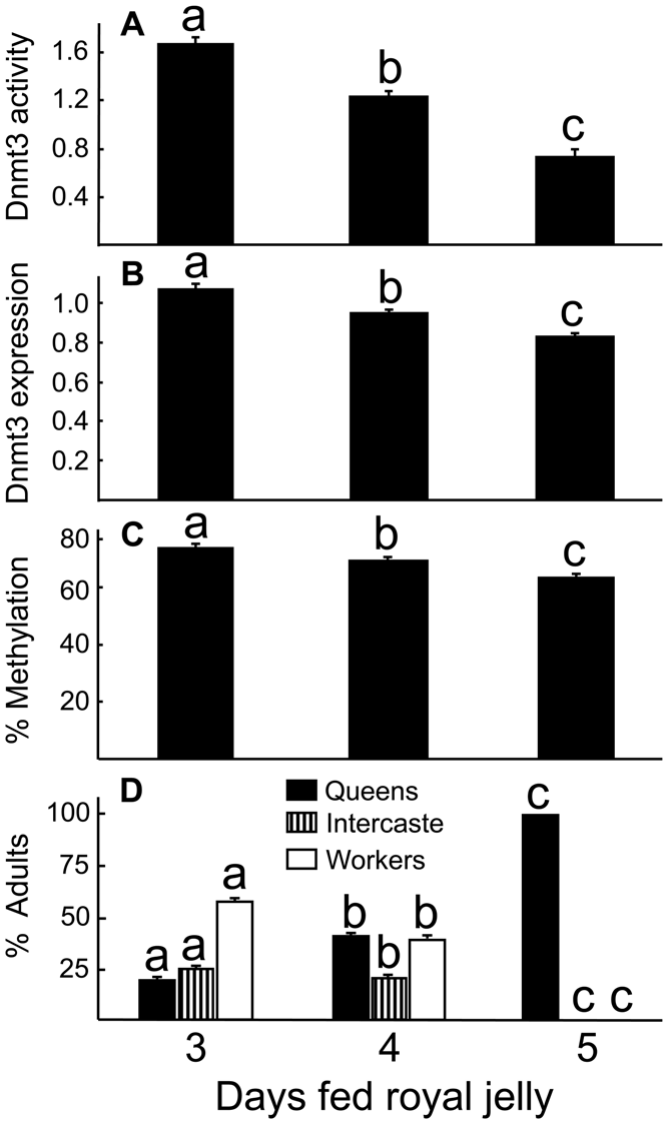
Effect of feeding larvae with 3, 4, or 5 days of royal jelly. Shown are Dnmt3 enzyme activity in mmol/min (A), Dnmt gene expression (B) relative to a reference gene *calmodulin*, and percentage of methylation in the gene *dynactin p62* (C) in 6-day old larvae and percentage of adults classified as queens, intercastes, or workers (D). Different letters on top of bars indicate significant difference (P<0.05) among the treatments with Fisher's Protected Least Significant Difference after analysis of variance showed a significant overall effect (P<0.05, A–C), or contingency table analysis with X^2^ (P<0.05) as the test statistic (D). In D, all comparisons are among different days, not among different castes within a single day. Data for Dnmt3 activity (A) were transformed by square root transformation; Dmnt3 expression (B) and percent methylation (C) were analyzed after arcsin transformation and presented here after transformation.

Larvae fed with royal jelly for different durations resulted in many different physiological changes. Larvae fed RJ for a longer duration resulted in significantly lower Dnmt3 activities (Fig. 2A), significantly lower Dnmt3 mRNA expression (Fig. 2B), and significantly lower rates of methylation for the gene *dynactin p62* (Fig. 2C) (P<0.05 in all tests, ANOVA). These changes were also correlated with a significantly increased proportion of queens produced, and significantly decreased proportions of both intercastes and workers in each group (P<0.05, Fig. 2D). Analysis of each individual methylation site indicated that at almost half of the CpG sites (4 out of 10), increased RJ feeding duration were associated with significantly lower rates of methylation (Table 1A).

### Effect of cell-size on caste differentiation

**Figure 3:**
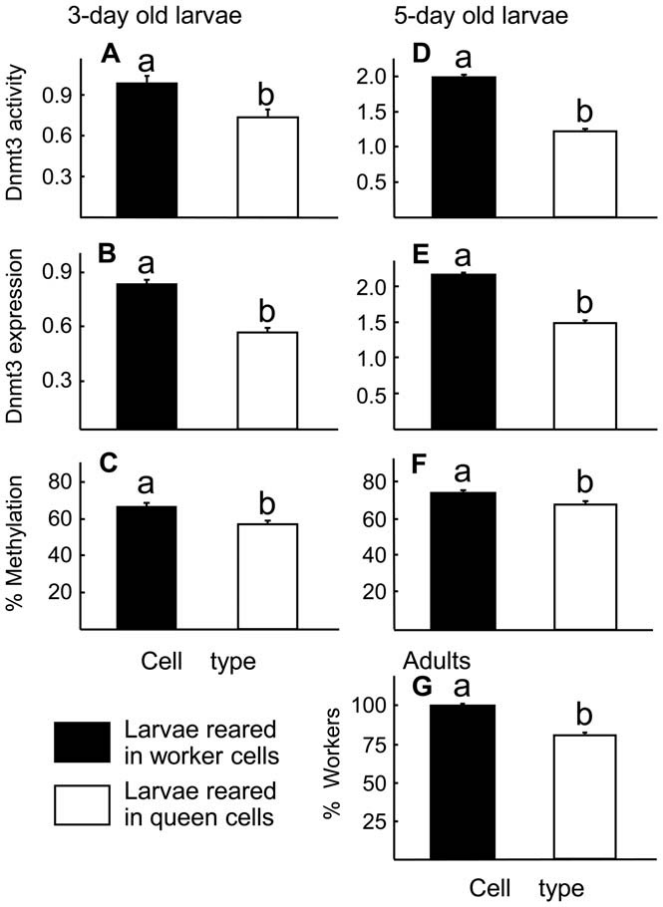
Effect of cell size on honey bee larvae. Shown are Dnmt3 enzyme activity in mmol/min (A, D), Dnmt3 gene expression (B, E) relative to a reference gene *calmodulin*, percentage of methylation in the gene *dynactin p62* (C, F) in 3-day (left) and 5-day old larvae (right), and percentage of adults emerged as workers (G). The non workers here (19% for queen cells) were all intercastes. Data analysis and transformation were the same as Fig. 2.

When assayed at 3 and 5 days of age, larvae reared in queen cells showed significantly lower levels (P<0.05 for all parameters, ANOVA) of Dnmt3 activity, mRNA expression, and overall rate of methylation (Fig. 3A–F, respectively). The proportion of workers was also significantly higher for larvae reared inside worker cells than those reared in queen cells (100% vs. 81%, Fig. 3G). The 19% non-workers in Fig. 3G were all intercastes and no queens were produced, despite of all the physiological differences observed. Individual analysis of each methylation site indicated that less than half of the CpG sites (4 out of 10) showed significantly reduced rates of methylation in queen-cell reared larvae compared to those reared in worker-cells in 3 day old larvae. However, in 5 day old larvae, more than half of the CpG sites (6 out of 10) showed significantly reduced rates of methylation in queen-cell reared larvae compared to those reared in worker-cells (Table 1B).

## Discussion

The results of this study demonstrate that 1). Increasing the duration of royal jelly caused a graded response in decreased methyltransferase enzyme activity, decreased methyltransferase gene expression, and decreased methylation in the gene *dynactin p62*, which in turn resulted in a significant increase of queens and reduction of workers or intercastes; 2). Cell size also contributed to caste differentiation; the larger queen cells resulted in lower methyltransferase enzyme activity, lower methyltransferase gene expression, and lower percentage of methylation in the gene *dynactin p62*, resulting in a significantly lower percentage of workers produced and 3). Diet type and cell size, though both acting on caste differentiation through modulation of methyltransferase activity, its gene expression and rates of methylation of *dynactin p62*, affected different CpG methylation sites. These results suggest that cell size, a previously largely ignored factor, also contributes to caste differentiation.

Kucharski et al. [12] reported that Dnmt3 silencing caused both decreased methylation levels and increased frequency of queen phenotype. They also studied 10 CpG sites in *dynactin p62* gene and discovered decreased overall levels of methylation across these 10 sites. In our study, we designed three primers to encompass 14 CpG sites distributed more broadly across nearly all exons (Fig. 1), except exon 6 (which was designated as exon 7 in both Kucharski et al. [12] and Wang et al. [11] papers; the current NCBI uses updated genomic information and annotates *dynactin p62* as having 9 exons instead of 8 exons, causing a shift in exon numbers in this study compared with the two mentioned studies) and exon 9 which we did not include. Our study manipulated the days larvae were fed with RJ and showed results consistent with Kucharski et al. [12]. Larvae fed RJ for the longest duration (3 days of RJ inside colony+2 days of RJ in laboratory) showed the lowest Dnmt3 activity, lowest Dnmt3 gene expression, lowest overall methylation of the gene *dynactin p62* and the lowest proportion of workers or intercastes (Fig. 2). All (100%) emerged adults were queens in this treatment (Fig. 2D). Those fed RJ for 4 days (3 days of RJ inside colony+1 day of RJ in the laboratory+1 day of WJ in laboratory) showed intermediate levels in all the measured variables and intermediate proportions of the three castes. Those fed RJ for 3 days (3 days of RJ inside colony+2 days of WJ in laboratory) showed the highest Dnmt3 activity, Dnmt3 gene expression, overall rates of methylation of the gene *dynactin p62* and the highest proportions of workers and intercastes. These results suggest that the response to the duration larvae are provided with RJ is graded, and is consistent with earlier results that the queen-worker difference is a continuum and not an on/off phenomenon [5].

It is well known that worker larvae up to 3 days old are still able to develop into a largely queen phenotype [5]. We therefore expected to observe no differences between the two groups of 3 day old larvae reared in different sized cells, because both received the same diet. Instead, we observed that in 3 day old larvae, all measured responses (Dnmt3 activity, expression, and percent methylation) were significantly different between the queen-cup and worker-cell reared larvae (Fig. 3 A–C). This suggested that even at 3 days old, the larvae somehow detected differences in their rearing environment, even though larvae are still relatively small (compared to worker cell size). These differences did not become strikingly larger when larvae were 5 days old (Fig. 3D–F). Space restriction is the only explanation for the observed differences, because we removed the gravity factor (in the colony, queen cells are oriented differently from worker cells) in the laboratory larvae-rearing setting.

In addition to Dnmt3 expression and rate of methylation in the gene *dynactin p62*, we also measured enzyme activity of Dnmt3 (Fig. 2A, 3A and 3D). Visual comparison of Fig. 2A vs. 2B suggests that Dnmt3 enzyme activity represents a more sensitive assay compared to Dnmt3 expression, because the differences between the 3 and 5 “Days fed royal jelly” were more striking in the Dnmt3 enzyme activity than the Dnmt3 expression levels. However the two measurements become similar in the cell type experiment (Fig. 3A, B, and D, E). These results suggest that the two factors (diet type and cell size) affect Dnmt3 activity and its gene expression differently.

Results in Table 1 suggest that diet type and cell-size affected different CpG sites. Diet type significantly affected the methylation rates of *dynactin p62* at sites 4, 5, 8, and 14, while cell-size affected sites 1/2, 3, 4, 7, 8 and 10. Only site 4 and 8 were affected by both factors and site 6 and 11/12/ were not affected by either one. This suggests that the two factors may act in two different pathways. Distinct genes might be differentially methylated when these two factors are manipulated, but this needs further experimental confirmation.

Results from Fig. 2D indicate that changing the larval diet from 3 days of RJ to 5 days of RJ decreased workers from 57% to 0%, suggesting a complete switch-over due to dietary manipulation. Fig. 3G shows that 100% of larvae when residing in worker cells became workers, while 81% became workers when residing in queen cups. The other 19% were of intercastes with no queen produced. The change of proportion of workers occurred solely due to cell size difference, because both groups were fed with the same diet (WJ harvested from 3 day old larvae). This represents a 19% reduction of workers, about one fifth of the effect of the dietary effect (that is if we ignore the fact that intercastes are not queens). These results suggest that even though cell-size can influence caste differentiation, its effect is weaker compared to the dietary effect.

DNA methylation has become a major focus in honey bee research [12], [15], [16] after its initial discovery in the system [11]. Our study measured for the first time the activities of methyltransferase Dnmt3, and provides evidence that cell types also affect DNA methylation, resulting in differences in the queen-worker development. Further studies are needed to understand how information about cell-size difference is transduced into physiological changes in the two castes.

## Materials and Methods

The Western honey bee, *Apis mellifera*, was used throughout this study. The honey bee colonies were maintained at the Honeybee Research Institute, Jiangxi Agricultural University, Nanchang, China (28.46°N, 115.49°E), according to standard beekeeping techniques. All experiments were conducted in a single colony to ensure genetic similarity among the larvae used.

### Effect of diet on caste differentiation

Honey bee larvae were reared inside plastic queen cups inside the colony for the first three days. These larvae did not require grafting because eggs were laid directly into queen cups by confining the queen inside a special cage. These queen cups can then be detached and used for royal jelly (RJ) production [17]. After the first three days, the larvae were divided into three groups and reared in the same queen cups in an incubator (35°C and 78±7% RH). The three groups received either newly harvested RJ (harvested the same day from the same colony) for 2 days (5 day RJ), or RJ for the first day, then freshly collected worker jelly from old larvae (WJ) (4 day RJ), or WJ only for 2 days (3 day RJ). Larvae were transferred every 12 hrs to queen cups with new food (200 µl per cup). On day 6, four samples each were taken for determining enzyme activity, gene expression of Dnmt3, and rate of methylation, with each sample containing 10 larval heads. The experiment was replicated in two separate trials (1060 larvae per trial), yielding 8 samples (each with 10 larval heads) per group.

### Effect of cell-size on caste differentiation

Honey bee larvae were reared inside plastic queen cups or worker cells inside an incubator (35°C, 78±7% RH) from day 1 (within 24 hours of larval hatching). Each cell was primed with 200 µl of freshly collected WJ (harvested from 3 day old worker larvae the same day) before the larva was transferred into it. Queen cups were of the same type as the first experiment. Worker cells were on pieces of natural beeswax comb (each about 398×152 mm). Larvae were transferred every 8 hrs to queen cups or worker cells with new food. Larvae were sampled on day 3 (whole larvae) and day 5 (heads only) for the same three parameters as the first experiment, with N = 8 samples for each parameter (Dnmt3 activity, gene expression, and rates of methylation) with each sample containing 10 larvae or heads. On day 6, mature larvae were transferred to 6-cell tissue culture plates (Costar, NY, USA) lined with a piece of Kimwipe and kept in an incubator (35°C and 78±7% RH) for pupation [18]. The overall emergence rate (1 day old larvae to adults) was 56.6%. We sampled 80 adult bees per group to score their morphological characteristics. We reared 1400 larvae for each trial and the experiment was replicated in two different trials.

### Experimental Details

#### Harvest of RJ and WJ

RJ was produced according to standard practices in China [19]. Briefly, the queen was separated from the rest of the colony by a queen excluding board. Queen cups with young larvae (one day old) were introduced into the colony and allowed to be fed by workers for 2 days. Larvae were then removed and fresh RJ removed with a spatula and stored at 4°C until use. To acquire worker jelly from old larvae, we first carefully removed 3 day old larvae by using either a grafting tool or a pair of forceps, then removed the WJ using a spatula. In experiment 1, both RJ and WJ came from the same colony as the experimental larvae.

#### Measurement of DNA methyltransferase 3 activity

Nuclear extract of larvae was prepared following the protocol of EpiQuik™ Nuclear Extraction Kit (Epigentek, Brooklyn, NY, USA). Dnmt3 activity was tested according to the procedure of EpiQuik™ DNA Methyltransferase Activity/Inhibition Assay Kit (Epigentek, Brooklyn, NY, USA). Absorbance at 450 nm was read on a Multiskan MK3 Microplate Reader (Thermo Fisher Scientific Shanghai Instruments Co Ltd, Shanghai, China).

#### Measurement of DNA methyltransferase 3 gene expression

Total RNA was isolated according to Trizol/QIAGEN RNeasy procedure (QiaGen, USA). Synthesis of cDNA was performed using Revertra Ace (Toyobo, Japan) according to manufacturer's instructions. Honey bee *calmodulin* gene was used as reference. Primers for *Dnmt3* and *calmodulin* were from Ying et al. [11] and Kucharski et al. [12], respectively. The cycling parameters were: 94°C, 2 min, 40× (95°C, 10 sec, 60°C, 15 sec, 72°C, 15 sec), and 72°C, 10 min. RotorGene v6.0 software (Corbett Research, Australia) was used for quantitative data extraction.

#### Measurement of methylation status of dynactin p62

Total DNA was isolated according to the protocol of the PUEX Animal Genomic DNA Extraction Kit (Cat. No. 3625031 PUEX, Beijing, China). Purified DNA (>50 ng per sample) was then sent to CapitalBio (Beijing, China) for quantitative methylation analysis, their protocol was published in Wang *et al.* [20]. Briefly, global methylation rates were determined with the Methylamp Global DNA Methylation Quantification Ultra Kit (Epigentek, New York, NY, USA). For individual methylation site determination, genomic DNA was first bisulfite-treated with the Methylamp DNA Modification Kit (Epidgentek). Then quantitiative methylation analsis was done by the Sequenom MassRARRAY Platform (CapitalBio, Beijing, China). The system uses matrix-assisted laser desorption/ionization time-of-flight (MALDI-TOF) mass spectrometry combined with RNA based-specific cleavage (MassCLEAVE).

The following are the sequences of three primers used for *dynactin p62* (XP_001121083):

*dynactin*_1-10F: aggaagagagGAAGATTTATTTTTGTAGGTATTGTT
*dynactin*_1-T7R: cagtaatacgactcactatagggagaaggctTTACTCTTAAAAATACATATCCAACTC
*dynactin*_2-10F: aggaagagagAATTAGTTATAGGAGGTTGGTTTGAA
*dynactin*_2-T7R: cagtaatacgactcactatagggagaaggctAATTCCTCTACTTCTATACTAACAACAA
*dynactin*_3-10F: aggaagagagATTAGAAGTAAGGATTGTTATTTGTGAA
*dynactin*_3-T7R: cagtaatacgactcactatagggagaaggctTCATCATATTCAACAACATCATCTCT

#### Measurement of morphometrics

After adult emergence, each individual was measured for their emergence weight (accurate to 1 mg). Other morphological characteristics were measured using a Panasonic Color CCTV camera (WV-GP240, Suzhou Co Ltd) and Computer Graphical Analysis System (T20080324-X0690-Z, Beijing TianHong Precision Instrument Technology Co. Ltd, China). These included length of body, length and width of hindwing, proboscis length, and the length of the third tergum. Ovaries from queen, queen-like, and worker bees were dissected under a dissecting scope and counted according to Li and Xiao [21]. We also noted the presence or absence of corbicula on the hindleg of each adult bee. Workers, intercastes and queens were identified based on number of ovarioles per ovary and presence/absence of corbicula, as previously described [12].

#### Data analysis

Results from physiological measurements were analyzed by analysis of variance (ANOVA) using StatView (v 5.01, SAS Institute, Gary, NC, USA). Multiple comparisons of the means were carried out using Fisher's Protected Least Significant Difference only after ANOVA showed significant effect (P<0.05). To meet the requirements of ANOVA, Dnmt3 gene expression data were transformed by square root, and rates of methylation were transformed by arcsin, before performing ANOVA. When ANOVA required data transformation, the data presented in Figures and Tables were after transformation. Means and their standard errors are presented. For the proportion of workers produced from different treatments, contingency table analysis and Chi-square statistic was used.
